# The Potential for Bio-Mediators and Biomarkers in Pediatric Traumatic Brain Injury and Neurocritical Care

**DOI:** 10.3389/fneur.2013.00040

**Published:** 2013-04-26

**Authors:** Patrick M. Kochanek, Rachel P. Berger, Ericka L. Fink, Alicia K. Au, Hülya Bayır, Michael J. Bell, C. Edward Dixon, Robert S. B. Clark

**Affiliations:** ^1^Safar Center for Resuscitation Research, Department of Critical Care Medicine, University of Pittsburgh School of MedicinePittsburgh, PA, USA; ^2^Child Advocacy Center, Department of Pediatrics, Children’s Hospital of Pittsburgh of UPMCPittsburgh, PA, USA; ^3^Children’s Hospital of Pittsburgh of UPMC, Department of Critical Care Medicine, University of Pittsburgh School of MedicinePittsburgh, PA, USA; ^4^Pittsburgh Center for Free Radical and Antioxidant Health, Department of Environmental and Occupational Health, University of Pittsburgh School of MedicinePittsburgh, PA, USA; ^5^Brain Trauma Research Center, Department of Neurological Surgery, University of Pittsburgh School of MedicinePittsburgh, PA, USA

**Keywords:** cerebrospinal fluid, abusive head trauma, shaken baby syndrome, neuron specific enolase, S100β, GFAP, myelin basic protein, UCH-L1

## Abstract

The use of biomarkers of brain injury in pediatric neurocritical care has been explored for at least 15 years. Two general lines of research on biomarkers in pediatric brain injury have been pursued: (1) studies of “bio-mediators” in cerebrospinal fluid (CSF) of children after traumatic brain injury (TBI) to explore the components of the secondary injury cascades in an attempt to identify potential therapeutic targets and (2) studies of the release of structural proteins into the CSF, serum, or urine in order to diagnose, monitor, and/or prognosticate in patients with TBI or other pediatric neurocritical care conditions. Unique age-related differences in brain biology, disease processes, and clinical applications mandate the development and testing of brain injury bio-mediators and biomarkers specifically in pediatric neurocritical care applications. Finally, although much of the early work on biomarkers of brain injury in pediatrics has focused on TBI, new applications are emerging across a wide range of conditions specifically for pediatric neurocritical care including abusive head trauma, cardiopulmonary arrest, septic shock, extracorporeal membrane oxygenation, hydrocephalus, and cardiac surgery. The potential scope of the utility of biomarkers in pediatric neurocritical care is thus also discussed.

## Introduction

Serum or cerebrospinal fluid (CSF) biomarkers of brain injury have been suggested as possible diagnostic adjuncts in neurocritical care since the groundbreaking work from the laboratory of Per Vaagenes (Kjekshus et al., 1980; Vaagenes et al., 1980, 1984, 1986, 1987, 1988; Bohmer et al., 1983; Vaagenes, 1986) in experimental and clinical cardiac arrest (CA), anoxic brain injury, stroke, and open heart surgery over 30 years ago. Earlier work in the 1960s concluded that serum creatine kinase, aspartate aminotransferase, and lactate dehydrogenase were not helpful as brain injury biomarkers (Dubo et al., 1967). However, in an often overlooked but remarkably prescient series of reports, his team used creatine phosphokinase brain band (CPK-BB) as the primary biomarker, along with CSF lactate dehydrogenase and aspartate aminotransferase and found that levels of these markers increased in CSF across these conditions. The studies included canine models of CA, and patients with CA, anoxic brain injury, cardiac surgery, or stroke. This was remarkably translational work for the early 1980s. They also explored the possible theragnostic utility of CSF biomarkers in patients treated with hypothermia vs. normothermia after CA (Vaagenes, 1986). Vaagenes called this approach “*a chemical biopsy of the brain*” and indicated that it may be clinically useful in prognosticating or in determining appropriate “levels of care.” Although the development of brain injury biomarkers is certainly challenging, it is unclear why we did not listen more carefully to him and further pursue this line of investigation 30 years ago.

The use of biomarkers of brain injury in pediatric neurocritical care has been explored for at least 15 years. We believe that studies in pediatric populations and applications are essential. Although in many cases, serum biomarkers of brain injury perform similarly in adult and pediatric applications, it is important to recognize that some biomarkers, show important age dependent differences in normal values in biological samples including CSF or serum. The most well recognized in this regard is S100B which exhibits high levels during infancy. These developmental increases also appear to be somewhat variable in magnitude and thus mandate that need for age matched controls when using this biomarker (Portela et al., 2002; Gazzolo et al., 2003). Thus, it is important to include the full spectrum of pediatric age groups when testing new pediatric biomarkers. Similarly, some disease processes exhibit age dependent second injury mechanisms such as the propensity toward neuronal apoptosis early in development. Brain injury biomarkers can have unique applications in pediatric traumatic brain injury (TBI) such as in detection of clinically silent brain injury in abusive head trauma (AHT) (Berger et al., 2006b). Finally, the nature of brain injury and its time course vary greatly across the spectrum of insults seen in pediatric neurocritical care and it is likely that the serum or CSF biomarker signature generated for each insult will differ. Our prior study of serum brain injury biomarker levels in infants and children across neurological diseases in the PICU confirmed that fact (Berger et al., 2006a), and represents an initial step in this regard. These issues mandate the development and testing of brain injury bio-mediators and biomarkers specifically in pediatric applications.

In this review, we will begin with studies of bio-mediators and biomarkers of brain injury in pediatric TBI and then broaden the discussion to other key disease processes associated with brain injury in the pediatric intensive care unit (ICU). This includes AHT, CA, and other pediatric neurocritical care conditions where brain injury biomarkers are showing promise.

### Early studies on bio-mediators and biomarkers of brain injury in pediatric TBI

**Figure 1:**
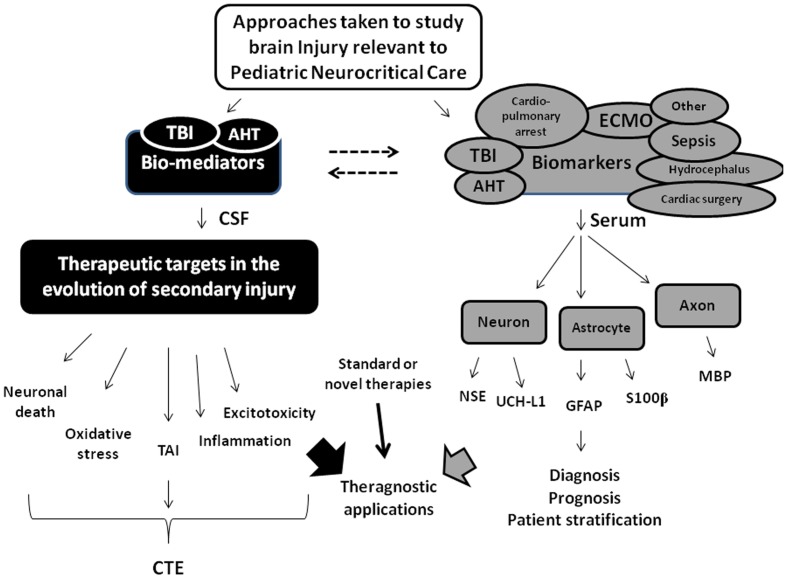
**Overview of the approaches taken by our research team and others in the application of bio-mediators and biomarkers of brain injury to study pediatric neurocritical care**. Two basic strategies have been utilized, namely (1) studies using bio-mediators to study evolution of secondary injury and define new therapeutic targets (shown in black), and (2) studies of biomarkers largely of structural origin released from injured or dying cells representing three major cellular components in the brain (i.e., neurons, astrocytes, and axons) to serve as diagnostic or prognostic adjuncts. For the studies of bio-mediators of secondary injury, this approach has been largely carried out in traumatic brain injury (TBI) using assessment of cerebrospinal fluid (CSF) that is drained as part of standard of care to reduce intracranial pressure. In those studies, five major secondary injury pathways including neuronal death, oxidative stress, traumatic axonal injury (TAI), inflammation, and excitotoxicity have received the most investigation using this approach. These pathways can ultimately lead to long-term disabilities and/or chronic traumatic encephalopathy (CTE). For the studies of biomarkers (shown in gray), although many biomarkers have been examined, five have been used in the majority of studies including the neuronal markers neuron specific enolase (NSE) and ubiquitin C-terminal hydrolase-L1 (UCH-L1), the astrocyte markers S100β and glial fibrillary protein (GFAP), and the axonal injury marker myelin basic protein (MBP). Both approaches have theragnostic applications. The dotted lines reflect that recognition that some “bio-meditros” can serve as biomarkers and vice versa. Please see text for additional details. AHT, abusive head trauma; ECMO, extracorporeal membrane oxygenation.

Bell et al. (1997b) examined CSF levels of the cytokines interleukin-6 (IL-6) and IL-10 in infants and children after severe TBI (Glasgow coma scale score < 8) and reported marked increases of both vs. controls. The levels of IL-6 in CSF were similar to the levels of IL-6 in serum in separate children with septic shock (Bell et al., 1997a), highlighting the surprising magnitude of the “inflammatory response” in brain after TBI, and suggesting that IL-6 might be useful as a biomarker of brain injury after TBI. Most of the early work on biomarkers of brain injury in children focused on TBI which is logical given its prevalence in children, and the availability of CSF as a biological sample source with the use of CSF diversion in the treatment of patients with severe TBI including AHT (Kochanek et al., 2012a,b). In general, two lines of research have been pursued: (1) studies of “bio-mediators” in CSF of children after TBI to explore the secondary injury cascade in an attempt to identify potential therapeutic targets and (2) studies of the release of structural proteins into the CSF, serum, or urine in order to diagnose, monitor, and/or prognosticate in patients with TBI. Although there is overlap between what constitutes a bio-mediator vs. a biomarker, the use of this construct to categorize studies is helpful. Among those studies, we published several seminal reports such as the aforementioned study on IL-6, the first use of CSF biomarkers to examine the molecular footprints of apoptotic neuronal death (Bcl-2, cytochrome *c*) after pediatric TBI (Clark et al., 2000; Satchell et al., 2005), and the first studies targeting use of serum biomarkers to aid in making the diagnosis of silent brain injury in infants with AHT (Berger et al., 2006b, 2009). We will discuss these and other recent studies on bio-mediators and biomarkers in pediatric TBI. Finally, in 2006, Berger et al. (2006a) published a study on the potential utility of three different serum biomarkers [neuron specific enolase (NSE), S100β, and myelin basic protein (MBP)] in three common pediatric neurocritical care diseases, namely, TBI, AHT, and cardiopulmonary arrest. Subsequent to that publication, other groups have published promising reports on the potential utility of these and several other serum biomarkers to identify brain injury in important diseases encountered in the pediatric ICU including recent reports on septic shock, extracorporeal membrane oxygenation (ECMO), hydrocephalus, and cardiac surgery (Cengiz et al., 2008; Hsu et al., 2008; Bembea et al., 2011; Bhutta et al., 2012). The potential scope of the utility of biomarkers in pediatric neurocritical care will thus also be discussed. An overview of the topics addressed in this review is provided in Figure 1.

## Defining the Evolution of Secondary Damage in Pediatric TBI Using CSF “Bio-Mediators”

The concept that CSF could be used to assess bio-mediator substances involved in secondary injury mechanisms was suggested as early as 1949 as shown for the neurotransmitters acetylcholine and serotonin by Tower and McEachern (1949) and Sachs (1957) in studies focused on clinical TBI (reviewed by Hayes et al., 1992). An early review on the use of CSF bio-mediators of brain injury in pediatric TBI provided initial rationale for the use of this approach (Kochanek et al., 2000) and the potential value of this line of investigation has gained support. Although the control of intracranial hypertension after severe TBI is important to prevent secondary brain ischemia and herniation, recent studies have suggested the need for additional therapies targeting other mechanisms of secondary damage. A multi-center randomized controlled trial (RCT) of decompressive craniectomy in adults with severe TBI (Cooper et al., 2011) showed that despite better control of raised ICP with surgical decompression, outcomes were worse vs. medical management – which unlike surgery, may be treating both ICP and other secondary injury mechanisms. Similarly, Mehta et al. (2010) reported that despite highly successful control of ICP in infants with severe TBI, ∼50% of children <2 years of age still had unfavorable long-term outcomes. Taken together, these studies suggest that we need to define the pivotal molecular secondary injury pathways after TBI and target them with novel therapies. In a number of studies, we have used CSF bio-mediators for this purpose and suggest potential therapeutic targets. Selected studies are discussed below.

### Bio-mediators of neuronal death

Early work in TBI suggested that neuronal death resulted from necrosis either from the primary impact or secondarily from ischemia-reperfusion during intracranial hypertension (reviewed in Kochanek et al., 2000). However, brain tissue samples from adults with severe TBI suggested that the molecular footprints of apoptosis including Bcl-2, Bcl-xl, Bax, and/or cleavage of caspase-3 were detectable in the initial days after severe TBI (Clark et al., 1999). Subsequently, Clark et al. (2000) showed that increases in CSF levels of the anti-apoptotic protein Bcl-2 were seen in infants and children early after severe TBI and were correlated with survival. Satchell et al. (2005) followed up on that study and reported that CSF levels of the pro-apototic protein cytochrome *c* were increased in infants with severe TBI. Cytochrome *c* levels were increased vs. control, and associated with mortality and AHT as an injury mechanism. This suggested that victims of AHT might represent a specific target population for the use of anti-apoptotic therapies after pediatric TBI. However, it is difficult to determine whether effects attributed to AHT are occurring independent of young age, since most infants with severe TBI are victims of AHT. Caspase-3 levels are also known to be much higher early in development than in older children (or adults) based on pre-clinical studies (Yakovlev et al., 2001). In addition, female gender was associated with increased levels of cytochrome *c* after severe TBI in infants and children, which is consistent with the predominance of apoptosis as a cell death pathway after exposure of female vs. male neurons to neurotoxins in cell culture (Du et al., 2009). Increases in the CSF levels of cytochrome *c* after pediatric TBI and its association with AHT and female gender were confirmed in a study examining biomarkers of apoptosis vs. necrosis (Au et al., 2012). These studies suggest that apoptotic neuronal death may represent a therapeutic target in pediatric TBI, particularly in infants. Studies in experimental models of TBI suggested that levels of cleavage products of αII Spectrin might be able to aid in differentiating apoptotic vs. necrotic neuronal death mechanisms in TBI (Pike et al., 1998a,b). αII Spectrin is cleaved by either calpain during necrosis to 145 and 150 kDa degradation products or by caspase-3 during apoptosis to a 120 kDa degradation product. And this approach has also been used to estimate the time course and necrotic vs. apoptotic neuronal death in adult patients with severe TBI using CSF analysis (Pineda et al., 2007). A predominantly necrotic profile was seen in adults in the initial 5 days after injury. Monitoring markers of neuronal apoptosis after TBI thus could be particularly important in children and is an area for future clinical work. Studies in experimental models of TBI suggest that other neuronal death pathways may play important roles including autophagy, necroptosis, and pyroptosis (You et al., 2008; Du et al., 2009; Adamczak et al., 2012). Unique biomarkers of these processes are also needed to define the quantitative contribution of these pathways to the evolution of neuronal death after TBI and other disorders in neurocritical care. The studies on neuronal death mechanisms in pediatric TBI also highlight the fact that pediatric TBI includes the special condition of AHT. Although TBI resulting from motor vehicle accidents, falls, and other mechanisms seen in both children and adults produces heterogeneous pathologies, AHT adds considerably to this problem. In addition to routine TBI presentations such as contusion, subdural hematoma, or diffuse axonal injury, AHT often presents with unique pathologies (Ichord et al., 2007). For example, in many cases the CT findings are consistent with hypoxic ischemic encephalopathy (HIE) – possibly from apnea at the scene, delay in presentation, or cervical nerve root injury. In addition, AHT is often repetitive, and thus both acute and chronic TBI can be superimposed. Given these factors, serum and CSF bio-mediators and biomarkers of brain injury may have special value in AHT – as shown in a number of studies discussed in this review.

### Oxidative stress bio-mediators and biomarkers after pediatric TBI

Another mechanism that may represent an important therapeutic target is oxidative stress. Bayır et al. (2002) published the first comprehensive report on CSF markers of oxidative stress after severe TBI in children. Strong evidence for major losses of antioxidants such as ascorbate was seen along with increases in levels of markers of oxidative damage such as F2-isoprostane. During the initial week after injury, a progressive reduction of CSF levels of ascorbate was noted. This suggests ongoing oxidative stress in children after severe TBI. Mitochondrial dysfunction was shown to occur in brain tissue samples from patients with severe TBI (Verweij et al., 2000) and may serve as a key source for free radicals (Kagan et al., 2004, 2009). In an experimental model of pediatric TBI, selective oxidation of the mitochondrial lipid cardiolipin was seen early after injury, suggesting that mitochondria are an initial source of free radicals (Bayır et al., 2007). Given that cardiolipin oxidation is intimately linked to release of cytochrome *c*, oxidative stress may be critically linked to apoptotic neuronal death after TBI (Kagan et al., 2004, 2009). Antioxidants that target mitochondria may thus represent a logical strategy to target apoptotic neuronal death, which could be particularly important in pediatric TBI. Consistent with that hypothesis, mitochondrial targeting has shown impressive success in experimental models of pediatric TBI (Ji et al., 2012). CSF levels of antioxidants or oxidized cardiolipin, as assessed by oxidative lipidomics, might also represent excellent biomarkers for theragnostic use and merit future study (Tyurin et al., 2008; Kagan et al., 2009; Ji et al., 2012). Finally, in a theragnostic application focused on oxidative stress in pediatric TBI, mild therapeutic hypothermia markedly attenuated the increase in CSF levels of markers of oxidative stress, suggesting that hypothermia mitigates this mechanism in patients (Bayır et al., 2009).

### Bio-mediators of neuroinflammation

Another secondary injury mechanism that has been studied in pediatric TBI using CSF levels of bio-mediators is inflammation. Early work on CSF bio-mediators in pediatric TBI focused on inflammatory cytokines (Bell et al., 1997b). Subsequently, CSF levels of a number of inflammatory mediators were measured (Whalen et al., 2000; Amick et al., 2001; Robertson et al., 2001b; Han et al., 2002; Tong et al., 2004; Buttram et al., 2007; Fink et al., 2008; Salonia et al., 2010). A complete description of those studies is beyond the scope of this review; however, several points are noteworthy. First, severe TBI is consistently accompanied by a robust increase in CSF levels of cytokines and chemokines, particularly IL-6 and IL-8 (Bell et al., 1997a,b; Whalen et al., 2000; Amick et al., 2001; Buttram et al., 2007). Second, the inflammatory response is complex and contributes detrimental and beneficial effects depending on timing (Scherbel et al., 1999). It thus represents a perplexing therapeutic target. Third, multiplex technology has been useful to study cytokines and chemokines after TBI – allowing multiple mediators to be quantified in a single sample. A multiplex approach was used by Buttram et al. (2007) to test the effect of therapeutic hypothermia on CSF levels of cytokines and chemokines after severe TBI in children. Many inflammatory mediators were increased, but, surprisingly hypothermia had only modest effects on them. Cellular effectors of neuroinflammation include microglia, macrophages, and T-lymphocytes, and additional CSF markers are needed to determine if aspects of the inflammatory process can be therapeutically targeted in pediatric TBI.

### Biomarkers and bio-mediators of traumatic axonal injury

Traumatic axonal injury (TAI) represents a mechanism of secondary damage that has been receiving increased attention recently, particularly as new imaging modalities are revealing the scope of this process (Tong et al., 2004; Babikan et al., 2005; Galloway et al., 2008). TAI was once believed to represent largely a primary injury process, however, the importance of “secondary axotomy” resulting from calcium accumulation and mitochondrial failure in axons has gained support (Smith et al., 2013). In pediatric TBI, Su et al. (2012) reported on this pathway using CSF levels of MBP, showing marked and sustained increases in this biomarker. The levels were on the order of ∼1000-fold higher than control, suggesting a major contribution of TAI. Drugs targeting TAI have not been tested in pediatric TBI, although calpain antagonists, cyclosporine-A, and FK506 have shown promise in experimental models (Smith et al., 2013). In the study by Su et al. (2012) mild hypothermia did not reduce CSF levels of MBP after severe TBI. Therapies that target TAI are needed and theragnostic use of a TAI biomarker such as MBP is logical. Pre-clinical studies suggest that there may be more injury of unmyelinated than myelinated axonal fibers (Reeves et al., 2005), and thus, new CSF biomarkers of unmyelinated axons are needed.

Excitotoxicity is a widely accepted secondary injury mechanism early after TBI. It could underlie early post-traumatic seizures and subclinical status epilepticus which are important in infants and young children (Liesemer et al., 2011). Early work on excitotoxicity in pediatric TBI was carried out by Ruppel et al. (2001) who reported marked increases in CSF levels of glutamate and other excitatory amino acids after severe injury. The increases peaked early in most patients and were associated with AHT. Robertson et al. (2001a) showed that the increases in CSF glutamate were coupled to retaliatory increases in levels of the endogenous anti-convulsant adenosine. Excitotoxicity may also mediate synaptic injury and one study showed marked increases in CSF levels of the synaptic protein α-synuclein after severe TBI in children (Su et al., 2010). α-Synuclein levels were increased ∼5-fold early after injury vs. control and progressed to levels ∼10-fold higher over the first week. A hot area of research in TBI is in defining the link between acute injury and the development of chronic traumatic encephalopathy (CTE) (DeKosky et al., 2010). TBI is linked to a variety of neurodegenerative diseases including Parkinson’s disease (PD). Deposition of α-synuclein aggregates in Lewy bodies in PD suggests a link to this mechanism. Although this is an area of intense study in adults, particularly with mild repetitive TBI, there has been little study of this association in children. This is a vital area of future research for TBI biomarkers in pediatrics given the role of sports concussion and its link to CTE.

## Serum Biomarkers in Pediatric TBI and Cardiopulmonary Arrest

### Diagnosis and prognosis in TBI

Building on the work in CSF, studies on the potential application of serum biomarkers of brain injury in pediatric neurocritical care began to emerge and initially focused on TBI and cardiopulmonary arrest. These conditions represent two of the most common disease processes encountered in pediatric neurocritical care and were thus logical targets for initial work on serum biomarkers. For diagnostic and prognostic indications, the approach focused on the use of proteins that are largely structural in nature and as unique as possible to the CNS. Most of the studies in pediatrics have centered around five biomarkers, namely, the neuronal markers NSE and ubiquitin C-terminal hydrolase-L1 (UCH-L1), the astrocyte markers S100β and glial fibrillary protein (GFAP), and the axonal injury marker MBP. After demonstrating robust increases in NSE and S100β in CSF in infants and children with severe TBI (Berger et al., 2002), Berger et al. (2005) measured serum levels of NSE, S100β, and MBP in 100 infants and children with TBI in cases of varying severity. All three biomarkers showed significant increases vs. controls, with sensitivity and specificity of initial values, for example, of 71 and 64% (NSE) and 77 and 72% (S100β). This suggested promise for the use of these serum biomarkers as diagnostic adjuncts in severe pediatric TBI. The biomarkers were also increased in many children who presented with a GCS score of 15 suggesting possible utility across injury severities – although a comprehensive study of serum biomarkers in mild TBI in children remains to be completed. Fraser et al. (2011) also explored the potential use of the biomarker GFAP in severe TBI in children. Serum GFAP levels measured on day 1 correlated with Pediatric Cerebral Performance Category scores assessed at 6 months. GFAP may also thus represent a potentially useful serum biomarker of brain injury in pediatric neurocritical care. Finally, Berger et al. (2012) recently studied the potential utility of serum levels of UCH-L1 and αII-SDP in pediatric TBI. UCH-L1 and αII-SDP levels were increased in cases of moderate or severe (but not mild) TBI and were correlated with Glasgow outcome scale score. These correlations were stronger than those for NSE, S100β, and MBP. Taken together, these studies suggest promise for a number of serum biomarkers in diagnostic and prognostic applications across the injury spectrum in pediatric TBI.

### Diagnostic adjunct in AHT

An important subgroup of patients with TBI for potential utility of serum biomarkers is cases of AHT – particularly infants with mild injury in whom the diagnosis may be missed and confused with conditions such as colic or gastroenteritis (Jenny et al., 1999). Based on a series of reports, NSE and MBP were shown to be the most potentially useful as screening tools to identify brain injury in well-appearing infants with clinically silent AHT (Berger et al., 2006b). Those studies led to the development of an NIH-funded prospective case-control study on the use of serum biomarkers for this purpose that has now entered nearly 900 infants. Studies are also ongoing examining the utility of GFAP and UCH-L1 in this setting. We also carried out a study of the application of proteomics (2-dimensional gel electrophoresis) on the injury response in AHT and compared it to non-abusive mechanisms of TBI in infants and young children (Gao et al., 2007). Several unique aspects of the proteomic injury profile were seen in AHT, notably, a reduced acute phase response. Infants who were victims of AHT had CSF proteomic profiles with reduced levels of acute phase reactants such as haptoglobin and complement components vs. children with TBI from other causes such as motor vehicle accidents. This could reflect a delay in presentation or represent a consequence of repeated injury often seen in cases of AHT. We also used a Multiplex approach in an attempt to define a combination or panel of serum biomarkers with high sensitivity and specificity to detect silent brain injury in infants with AHT (Berger et al., 2009). In that study, vascular cellular adhesion molecule (VCAM) and IL-6, used together, could discriminate the AHT vs. control with a sensitivity and specificity of 87 and 90%, respectively, when evaluated in an appropriate pediatric population to target missed AHT. Further studies using combinations or panels of biomarkers are needed in AHT and across the relevant diseases in pediatric neurocritical care.

### Serum biomarkers of brain injury in pediatric cardiopulmonary arrest

We also carried out, to our knowledge, the first comparative study of serum levels of NSE, S100β, and MBP in critically ill infants and children after TBI, AHT, and cardiopulmonary arrest (Berger et al., 2006b). Distinct temporal profiles were seen for each of these conditions. TBI showed the largest acute increases in serum biomarker levels likely reflecting immediate damage from the primary injury. In cardiopulmonary arrest and AHT, delayed increases in the neuronal death marker NSE suggested its (or other neuronal death markers) potential utility for prognostic and theragnostic applications, and for the need to evaluate therapies targeting delayed neuronal death in cardiopulmonary arrest and AHT. Several studies in neonatal HIE from birth asphyxia have quantified serum biomarkers including S100β and NSE (Massaro et al., 2012; Roka et al., 2012). In 25 infants treated with either hypothermia or normothermia, serum S100β levels were lower in the hypothermia group and both S100β and NSE levels were higher in infants with worse outcome (Roka et al., 2012). In a larger study of 75 infants with neonatal encephalopathy and treated with hypothermia, S100β and NSE were again shown to be higher in patients with unfavorable outcome (Massaro et al., 2012). This suggests that these biomarkers are useful even if hypothermia is used in the treatment regimen. The astrocyte marker GFAP has also been shown to be increased in serum early after injury in neonates with HIE (Ennen et al., 2011). In preliminary studies, we reported use of three serum biomarkers NSE, S100β, and MBP as aids in prognostication in pediatric CA and observed outstanding performance based on receiver operator characteristic analysis (Fink et al., 2011). Several time points were employed, but 24 h values for NSE and S100β with cut points of 0.008 or 53.10 ng/mL exhibited high probability for classifying good vs. poor outcome in infants and children. This finding was seen despite the fact that there was heterogeneity in the etiologies of the arrests. Studies of the effect of mild hypothermia on biomarker levels and outcome are also ongoing including assessment of the efficacy of 24 vs. 72 h of hypothermia. Studies of the potential utility of UCH-L1 in pediatric CA are also ongoing. For additional discussion of serum biomarkers across adult and pediatric TBI and CA, the reader is referred to a prior review (Kochanek et al., 2000).

## Brain Injury Biomarkers Across Other Pediatric Neurocritical Care Diagnoses

There have been a number of new applications of brain injury bio-mediators and biomarkers in pediatric neurocritical care. We will highlight several recent and promising studies in this regard in septic shock, ECMO, hydrocephalus, and cardiac surgery.

### Serum biomarkers of brain injury in pediatric septic shock

Hsu et al. (2008) assessed serum levels of S100β, NSE, and GFAP over the initial week of presentation in 24 children with septic shock and reported substantial (∼10 and 20-fold) increases in S100β and NSE respectively, despite lack of focal neurological deficits on exam. However, continuous EEG revealed moderate to severe encephalopathy in the patients. Biomarker levels were low early after sepsis and peaked at 5–7 days, contrasting TBI or CA. It is unclear whether these increases reflect permanent or transient damage, are associated with any long-term neurological morbidity, or reflect increases from extracerebral sources (Redl et al., 2008). However, this study should serve as an excellent foundation for future work in this area.

### Serum biomarkers of brain injury during extracorporeal membrane oxygenation

Bembea et al. (2011) explored the use of plasma GFAP levels in 22 pediatric patients treated with ECMO for respiratory failure, cardiac failure, CA, or sepsis. Infants admitted to the ICU but without neurological injury served as controls. Seven infants treated with ECMO developed neurological complications including intracranial hemorrhage, cerebral edema, or brain death. Peak GFAP levels were ∼50-fold higher in these infants. Several temporal patterns were seen including progressive increases, or increases at single time points. The extracorporeal-CPR group was at highest risk for brain injury and increased plasma GFAP levels. A commentary on this report suggested the need for rigorous biokinetic analyses and the development of standardized assays for GFAP (Hayes et al., 2011). Children on ECMO are a perfect group for use of serum brain injury biomarkers given the difficulty in routine brain imaging during ECMO.

### CSF biomarkers in pediatric hydrocephalus

Cengiz et al. (2008) studied the application of CSF biomarkers of brain injury to another common diagnosis in pediatric neurocritical care, namely hydrocephalus. CSF levels of the neuronal injury marker cleaved-tau protein were assessed in 11 children with hydrocephalus requiring shunt placement or revision vs. values in controls. Cleaved-tau is a marker of neuronal damage or turnover formed by the proteolytic cleavage of the structural protein microtubule associated protein-tau (MAP-tau). Cleaved-tau CSF levels were increased in patients with hydrocephalus and correlated with duration of symptoms; ∼75% of the patients had signs of increased ICP before surgery. Tau-cleavage products are promising biomarkers of CTE and thus this study may represent a valuable early report on this topic in children relevant to TBI.

### Serum biomarkers of brain injury–theragnostic application in cardiopulmonary bypass

Finally, several groups have tested serum biomarkers of brain injury in the setting elective cardiac surgery in children (Abdul-Khaliq et al., 2000; Ali et al., 2000; Matheis et al., 2000; Lindberg et al., 2003; Lardner et al., 2004; Liu et al., 2009; Bhutta et al., 2012). Although a complete review of those studies is beyond the scope of this review, several studies have explored the theragnositc use of brain injury biomarkers after cardiac surgery in children. In an RCT of ketamine (2 mg/kg IV, *n* = 13) vs. placebo (*n* = 11) before surgery in infants, plasma levels of NSE, S100β, cytokines, and C-reactive protein were assessed (Bhutta et al., 2012). C-reactive protein levels were lower with treatment, although whether this reflected differences in brain injury was unclear. Treatment reduced injury as reflected by choline and glutamate plus glutamine/creatine levels assessed by magnetic resonance spectroscopy (MRS) in frontal white matter, but no differences between groups were seen on behavioral testing post-operatively. A combination of serum biomarkers with MRS may represent a useful theragnostic approach in acute brain injury. This strategy is being used to study the effect of 24 vs. 72 h of hypothermia in pediatric CA (Fink et al., 2011). Matheis et al. (2000) used serum levels of S100β to show increased oxidative injury after uncontrolled vs. controlled re-oxygenation after cardiac surgery in infants. Abdul-Khaliq et al. (2000) used S100β to study the effect of treatment with sodium nitroprusside in 25 neonates after cardiac surgery and reported reductions in serum levels of this biomarker with treatment. Similar approaches have been taken for other therapies after cardiac surgery in children including corticosteroids (Lindberg et al., 2003).

## Conclusions

It is an exciting time for biomarker development and exploration of bio-mediators in pediatric neurocritical care and rewarding that after over 15 years of work in this area, use of these tools may become standardized and incorporated into routine clinical use for diagnosis, prognosis and other aspects of patient management. Assessment of bio-mediators and biomarkers in CSF and serum is also helping to define therapeutic targets and provide theragnostic value in monitoring treatment efficacy. Brain injury biomarkers may also guide patient stratification for clinical trials – to help define the best sample for future RCTs or help show treatment effects. This could be important given the many failures of trials in TBI and the heterogeneity of this and other conditions in pediatric neurocritical care. We look forward to the development of point of care technology for brain injury biomarker applications in pediatric neurocritical care.

## Conflict of Interest Statement

The authors declare that the research was conducted in the absence of any commercial or financial relationships that could be construed as a potential conflict of interest.
